# Impact of Surgical and Anesthetic Procedures after Colorectal Cancer Surgery: A Propensity Score-Matched Cohort Study (The PROCOL Study)

**DOI:** 10.3390/medicina60081362

**Published:** 2024-08-21

**Authors:** Céline Kuoch, Lucillia Bezu

**Affiliations:** 1 Département d’Anesthésie, Chirurgie et Interventionnel, Gustave Roussy, FR-94805 Villejuif, France; 2 U1138 Metabolomics and Cell Biology Platforms, Gustave Roussy, Université Paris Saclay, FR-94805 Villejuif, France; 3 EuroPeriscope Group, ESA-IC, Onco-Anesthesiology Research Group, B-1000 Brussels, Belgium

**Keywords:** anesthesia, cancer, colorectal, surgery

## Abstract

*Background:* Surgical inflammatory pain decreases the innate and adaptive immune antitumor response and favors residual circulating tumor cells. *Objectives:* This study investigated whether minimally invasive surgeries (laparoscopic and robotic procedures), which are less painful and inflammatory, improved oncological outcomes after colorectal resection compared to laparotomy. *Methods:* This research was a single-center propensity score-matched study involving patients who underwent colectomy and rectum resection from July 2017 to December 2019. *Results:* Seventy-four laparotomies and 211 minimally invasive procedures were included. Minimally invasive procedures were associated with less blood loss (0 mL vs. 75 mL, *p* < 0.001), shorter length of stay (8 days vs. 12 days, *p* < 0.001), and fewer complications at 3 months (11.8% vs. 29.4%, *p* = 0.02) compared to laparotomies. No difference in overall survival (OS) and recurrence-free survival (RFS) at 3 years between groups was observed. Univariate Cox regression analyses demonstrated that age and ASA > 3 can negatively impact OS, while adjuvant chemotherapy can positively influence OS. pT3-T4 stage and postoperative pain could negatively influence RFS. Multivariate Cox regression analyses concluded that age (HR 1.08, *p* < 0.01) and epidural analgesia (HR 0.12, *p* = 0.03) were predictors for OS. Lidocaine infusion (HR 0.39, *p* = 0.04) was a positive predictor for RFS. *Conclusions*: Minimally invasive procedures reduce postoperative complications and shorten the length of hospital stay compared to major surgeries without improving prognosis. However, the administration of local anesthetics through neuraxial anesthesia or intravenous infusion could improve survival and decrease the occurrence of relapses.

## 1. Introduction

Colorectal cancer is the second most deadly cancer worldwide, despite screening strategies [1]. Early surgical resection of the primary tumor remains the best curative treatment for this malignant disease. Major surgical procedures are performed depending on the size and location of the tumor, such as colectomy, proctocolectomy, or abdominoperineal resection [2]. However, due to pain and inflammation, surgery induces an immunosuppressive response, including the synthesis of protumor cytokines and the secretion of glucocorticoids [3]. This surgical stress impairs innate antitumor immune effectors and favors oncogenesis [4]. Furthermore, the manipulation of tumors by surgeons releases circulating tumor cells into the systemic circulation, which migrate to distant organs and generate metastases [5]. Some evidence suggests that the occurrence and number of metastases could be directly correlated to the intensity of the procedures [6]. Thus, despite its benefits, surgery alters the immune system already weakened by the disease and neoadjuvant therapy, favoring the circulation of residual malignant cells and their escape to immunosurveillance.

Since the 1990s, laparoscopic removal of colon or rectum tumors has progressively replaced the laparotomy approach [7,8]. In the 2000s, robotic procedures were implemented to minimize tissue damage and assist clinicians with surgical precision and control [9,10,11]. Published trials suggest better short-term outcomes after minimally invasive surgery, with fewer postoperative complications and shorter hospital stays [12]. However, the impact of minimally invasive surgery, including both laparoscopic and robotic surgery, on long-term outcomes compared to major open procedures remains unclear.

The type of procedure is not the only factor that seems to influence surgical outcomes. Ample preclinical data support that local anesthetics injected during oncological procedures could improve survival by inducing direct cytotoxic effects on residual tumor cells and promoting antitumor immune memory [13,14,15,16,17]. However, the results of clinical trials have not yet been conclusive. Among 10 published clinical trials, six retrospective and one prospective study observed significantly better overall survival (OS) and fewer relapses after the use of neuraxial anesthesia during colorectal cancer surgery [18,19,20,21,22,23,24,25,26,27]. Whether local anesthetics may be a predictor of survival after colorectal surgery remains an open question.

We hypothesized that minimally invasive surgeries such as laparoscopic and robotic procedures, causing less tissue damage, pain, and inflammation, could be less immunosuppressive and improve oncological outcomes. Herein, we aim to compare minimally invasive procedures and laparotomy for colorectal tumor removal. The primary outcome of this retrospective single-center propensity score-matched cohort is to investigate long-term oncological outcomes. Secondary outcomes are to assess intraoperative and postoperative complications and determine the predictive factors of OS and recurrence-free survival (RFS) with the aim of guiding clinicians in their practice.

## 2. Materials and Methods

### 2.1. Study Design

This study is a French single-center propensity score-matched cohort study. Patients who underwent colon or rectum resection for cancer were included. Data were retrospectively collected from the database of a French center (Gustave Roussy Hospital, Villejuif, France). The study was approved by the Institutional Review Board of Gustave Roussy (IRB 2022-120, 29 March 2022). The study was performed according to the Declaration of Helsinki. The study followed the STrenghtening the Reporting of OBservational studies in Epidemiology (STROBE) checklist [28].

### 2.2. Eligibility Criteria

Patients (≥18 years old) who underwent partial or total resection of the colon or rectum (colectomy, rectum resection, proctocolectomy, abdominoperineal resection) for cancer through laparotomy procedures or minimally invasive surgery (laparoscopic or robotic interventions) between 1 July 2017 and 31 December 2019 were eligible. The surgical approach was determined according to the tumor size and experience of the surgeon.

### 2.3. Non-Eligibility Criteria

Patients were not included if they were lost to follow-up, palliative care, cancer stage IV (=metastasis stage), prophylactic surgery, surgery performed for recurrences, or for invasion by another cancer.

### 2.4. Data Collection

#### 2.4.1. Demographic Data

Demographic data included sex, age, weight, height, and body mass index (BMI).

#### 2.4.2. Preoperative Data

Preoperative data included: the American Society of Anesthesiology (ASA) classification, active tobacco and alcohol consumption, undernutrition (weight loss >10% in the last 6 months), co-morbidity (stroke, hypertension, diabetes, chronic obstructive pulmonary disease [COPD], cardiopathy, cardiac failure [the New York Heart Association, NYHA, classification], kidney insufficiency [>110 µmol/L for males and >100 µmol/L for females]), family history of colorectal cancer, medication (aspirin, angiotensin-converting enzyme inhibitor/angiotensin II receptor blockers [ACEi/ARB], beta-blockers, opiate, statin), enhanced recovery after surgery (ERAS) program, neoadjuvant anticancer therapy (chemotherapy, radiotherapy), biology (albumin, creatinine, carcinoembryonic antigen [CEA], hemoglobin, leukocytes), tumor site (left, transverse, right colon or rectum), and TNM stage.

#### 2.4.3. Intraoperative Data

Intraoperative data included duration of surgery, transfusion, blood loss, anesthetics (total intravenous anesthesia [TIVA], remifentanil, volatiles), analgesics (acetaminophen, nefopam, ketamine infusion, morphine, tramadol, patient-controlled epidural analgesia [PCEA], lidocaine infusion, Transverse Abdominal Plane [TAP]-block, local anesthetic infiltration).

#### 2.4.4. Postoperative Data

Postoperative data included pTNM, R-category, microscopic resection margin, analgesics (acetaminophen, nefopam, non-steroidal anti-inflammatory drugs [NSAIDs], tramadol, postoperative morphine, total morphine (intra- and postoperative period)), visual analog scale (VAS) postoperative day (POD) 1 and 2, chemotherapy, time between surgery and chemotherapy, immunotherapy, length of stay, complications (Clavien-Dindo classification [29]) at POD7 and at 3 months, CEA at 3 years, recurrences (local, nodes, metastases), time between surgery and recurrences, death, and time between surgery and death.

### 2.5. Outcomes

The primary outcomes were OS and RFS at 3 years between the laparotomy group and the minimally invasive surgery group. Secondary outcomes were the predictor factors of OS and RFS, and intraoperative and postoperative complications.

### 2.6. Definition

OS was defined as the time between surgery and death. RFS was defined as the time between surgery and recurrence (local, nodes, or metastases).

### 2.7. Statistical Analyses

Statistical analyses were performed with R software version 4.3.0 (21 April 2023, The R Foundation for Statistical Computing Platform). Missing data were imputed using multiple imputations. Data normality was assessed using the Kolmogorov-Smirnov test. Quantitative Gaussian data were compared using a Student’s *t*-test and expressed as mean (SD). Quantitative non-Gaussian data were compared with a Mann–Whitney U test and expressed as median [interquartile]. Qualitative data were compared using a chi-square test or a Fisher’s exact test and expressed as percentages. Statistical significance was assessed for a 95% Confidence Interval (CI) and *p* < 0.05. OS and RFS were compared using the log-rank test and analyzed using Kaplan–Meier curves. Predictor factors for OS and RFS were assessed using univariate and multivariate Cox Hazard models. Clinically relevant variables from the total cohort were implemented in the Cox Hazard Regression model. Univariate Cox Hazard model analyses were performed with the following variables: age, sex, BMI > 25 kg/m^2^, ASA > 3, pTNM, R-category, type of surgery, surgery duration > 6 h, blood loss > 350 mL, transfusion, intraoperative remifentanil > 2100 µg, intraoperative morphine > 10 mg, PCEA, lidocaine infusion, total morphine consumption > 145 mg (oral morphine equivalents [OME]), postoperative NSAID, postoperative tramadol, VAS within 3 POD, and adjuvant chemotherapy. The cut-off values of blood loss, remifentanil consumption, intraoperative morphine, and total morphine consumption correspond to the highest third interquartile range between both groups. Then, variables with *p* ≤ 0.05, or that may influence the outcomes, were selected for the multivariate analyses.

#### 2.7.1. Groups and Sub-Groups

Two groups were compared: the laparotomy group (Laparo) and the minimally invasive surgery group (Mini). In the case of conversion, patients were included in the laparotomy group. Subgroup analyses were performed to compare laparotomy and minimally invasive surgery for colon cancer removal or rectum cancer removal. Univariate and multivariate analyses were not performed in the “rectum” subgroup due to the lack of included patients. Subgroup analyses were also performed in the Mini group by comparing conventional laparoscopic (Conv) and robotic (Robot) surgery.

#### 2.7.2. Propensity Score Matching

Propensity score matching was used to balance both groups and decrease the risk of selection bias. The propensity score was calculated using a logistic regression model (glm) and with the library “matching” from R software (version 4.3.0). Preoperative covariates with SMD > 0.25 and/or that may impact the primary outcome were included for the propensity score: age > 70 years old, sex (female vs. male), BMI > 25 kg/m^2^, ASA > 3, neoadjuvant chemotherapy, CEA, T3-T4 (vs. T1-T2), N+ (vs. N0). Patients were matched at a ratio of 1:1 according to the propensity score and with a caliper width of 0.1.

## 3. Results

### 3.1. Patients’ Characteristics

**Figure 1:**
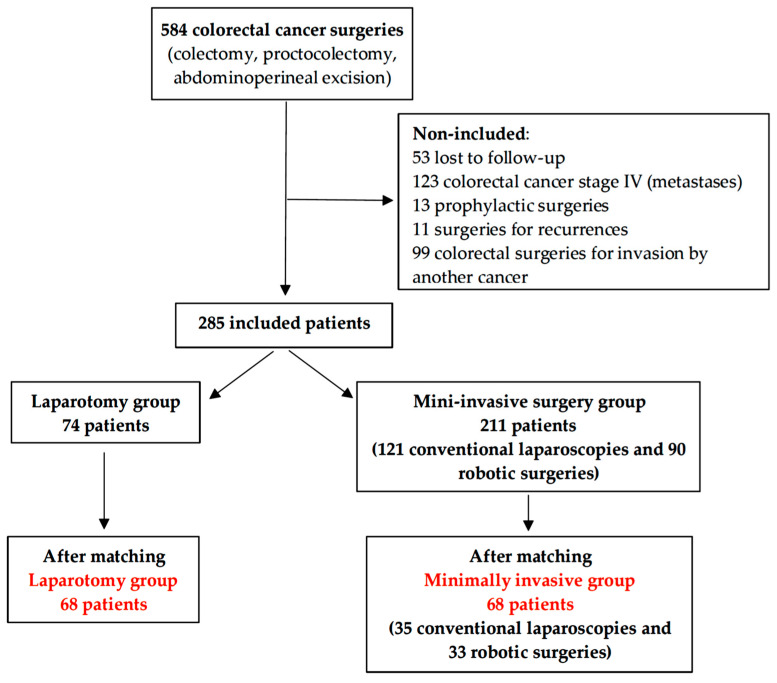
Flowchart before and after propensity score matching.

Between 1 July 2017 and 31 December 2019, 584 resections of colon or rectum primary tumors were performed in our center. Patients were not included if they were lost to follow-up, had stage IV cancer, prophylactic surgery, surgery for recurrences, or colorectal invasion by another cancer. Overall, 285 patients were included in the cohort: 74 patients in the Laparo group and 211 patients in the Mini group (121 conventional laparoscopies and 90 robotic surgeries) Figure 1. Before propensity score matching, baseline characteristics were not perfectly balanced between the groups. Fewer patients were overweight in the Mini group (43.1% vs. 58.1%, *p* < 0.001). Fewer patients received neoadjuvant chemotherapy in the Mini group (25.1% vs. 44.6%, *p* < 0.003). More rectum surgeries were performed in the Mini group (42.7% vs. 35.1%, *p* < 0.001). Fewer patients with T3-T4 stage and more patients with node invasion were included in the Mini group (29.9% vs. 44.6%, *p* = 0.03 and 70.1% vs. 55.4%, *p* = 0.006, respectively) Table 1. After propensity score matching, 68 patients were included in each group, and baseline variables chosen for the propensity score were balanced between both groups Table 1.

### 3.2. Intraoperative and Postoperative Variables

After propensity score matching, we observed that patients in the Mini group received more remifentanil (*p* = 0.02), more lidocaine infusion (*p* = 0.002), more morphine (*p* = 0.001), and had less PCEA (*p* < 0.001) compared to the Laparo group. Patients in the Mini group lost less blood (*p* < 0.001). The length of stay was significantly decreased in the Mini group (8 days [7–12.25] vs. 12 days [8.75–19], *p* < 0.001). Fewer complications (Clavien-Dindo classification) at 3 months were observed in the Mini group (11.8% vs. 29.4%, *p* = 0.02) Table 2.

### 3.3. Overall Survival

**Figure 2:**
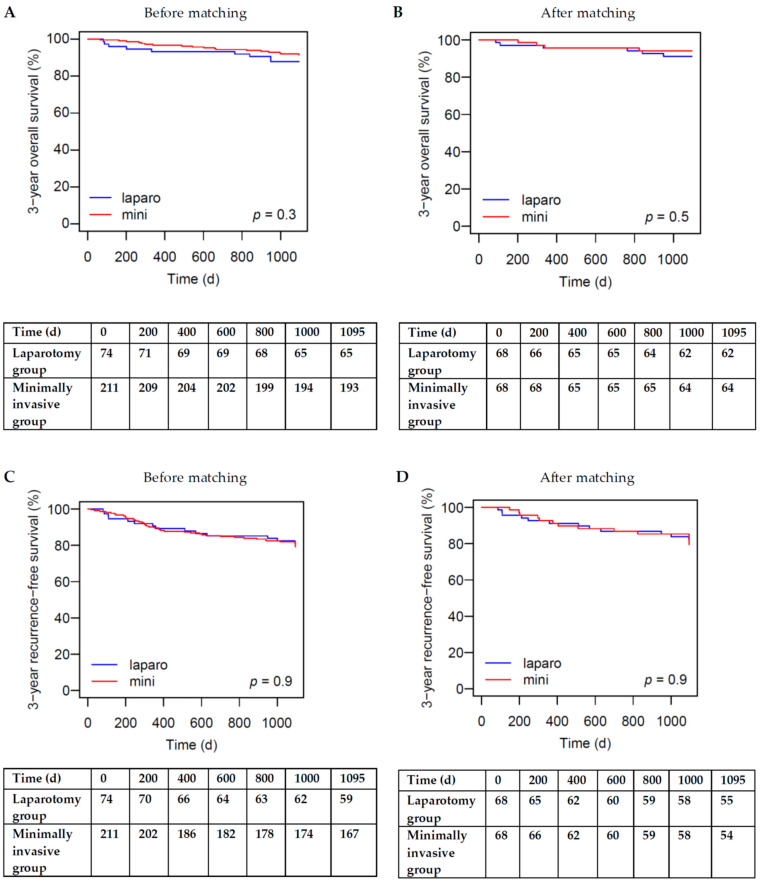
Three-year OS and RFS between laparotomy and minimally invasive surgery (laparoscopic and robotic surgery) for colorectal cancer resection. (**A**) Three-year OS before propensity score matching (log-rank test, *p* = 0.3). (**B**) Three-year OS after propensity score matching (log-rank test, *p* = 0.5). (**C**) Three-year RFS before propensity score matching (log-rank test, *p* = 0.9). (**D**) Three-year RFS after propensity score matching (log-rank test, *p* = 0.9).

Before propensity score matching, the median 3-year OS was not significantly different between the Laparo and Mini groups (331 days [109; 840] vs. 572.5 days [284.2; 880.2], *p* = 0.47). The Kaplan–Meier curves were not significantly different between the Laparo and Mini groups (log-rank test *p* = 0.3). The 3-year OS was 87.8% for the Laparo group and 91.5% for the Mini group (*p* = 0.36). After propensity score matching, the median 3-year OS was not significantly different between the Laparo and Mini groups (547 days [164.5; 820.8] vs. 318 days [273; 460.2], *p* = 0.91). The Kaplan–Meier curves were not significantly different between the Laparo and Mini groups (log-rank test *p* = 0.5). The 3-year OS was 91.2% for the Laparo group vs. 94.1% for the Mini group (*p* = 0.74) Figure 2A,B.

### 3.4. Recurrence-Free Survival

Before propensity score matching, the median 3-year RFS was not significantly different between the Laparo and Mini groups (350.5 days [134.5; 614] vs. 308.5 days [201; 578.2], *p* = 0.80). The Kaplan–Meier curves were not significantly different between the Laparo and Mini groups (log-rank test *p* = 0.9). The 3-year RFS was 79.7% for the Laparo group and 79.1% for the Mini group (*p* = 1). After propensity score matching, the median 3-year RFS was not significantly different between the Laparo and Mini groups (435 days [185.5; 709] vs. 340.5 days [225; 483.5], *p* = 0.77). The Kaplan–Meier curves were not significantly different between the Laparo and Mini groups (log-rank test *p* = 0.9). The 3-year RFS was 80.9% for the Laparo group vs. 79.4% for the Mini group (*p* = 0.83) Figure 2C,D.

### 3.5. Predictors for Overall Survival

Univariate Cox regression analysis demonstrated that age and ASA > 3 can negatively influence 3-year OS, while adjuvant chemotherapy can positively influence 3-year OS. These aforementioned factors and covariates that may influence OS (pT3-T4, PCEA, lidocaine infusion, and type of surgery) were included in the multivariate Cox regression analysis. Age was a negative factor for 3-year OS (HR 1.08, 95% CI [1.02–1.15], *p* < 0.01). Conversely, PCEA could be a positive factor for 3-year OS (HR 0.12, 95% CI [0.02–0.86], *p* = 0.03) Table 3.

### 3.6. Predictors for Recurrence-Free Survival

Univariate Cox regression analysis demonstrated that the pT3-T4 stage and pain with VAS > 3 during the three postoperative days could negatively influence 3-year RFS. These aforementioned factors and covariates that may influence RFS (pN+ stage, PCEA, lidocaine infusion, type of surgery, and adjuvant chemotherapy) were included in the multivariate Cox regression analysis. Lidocaine infusion could be a positive factor for 3-year RFS (HR 0.39, 95% CI [0.16–0.97], *p* = 0.04) Table 4.

### 3.7. Colon Cancer Surgery Versus Rectum Cancer Surgery

#### 3.7.1. Colon Cancer Surgery

The Kaplan–Meier curves were not significantly different between the Laparo and Mini groups for OS (before matching: log-rank test *p* = 0.3; after matching: log-rank test *p* = 0.6) and RFS (before matching: log-rank test *p* = 0.8; after matching: log-rank test *p* = 0.9) Figure S1.

Univariate Cox regression analysis demonstrated that age and VAS > 3 during the three postoperative days could negatively influence OS and RFS, respectively. Multivariate Cox regression analysis found that age was a negative factor for OS (HR 1.09, 95% CI [1.03–1.17], *p* < 0.01), and VAS > 3 during the three postoperative days could negatively impact RFS (HR 4.1, 95% CI [0.88–19.11], *p* = 0.07). Conversely, PCEA could be a positive factor for OS (HR 0.14, 95% CI [0.02–0.88], *p* = 0.04) and RFS (HR 0.21, 95% CI [0.05–0.98], *p* = 0.048) Tables S1 and S2.

#### 3.7.2. Rectum Cancer Surgery

The Kaplan–Meier curves were not significantly different between the Laparo and Mini groups for OS (before matching: log-rank test *p* = 1; after matching: log-rank test *p* = 0.4) and RFS (before matching: log-rank test *p* = 0.7; after matching: log-rank test *p* = 1) Figure S2.

### 3.8. Conventional Laparoscopy versus Robotic Surgery

The Mini group included 121 conventional laparoscopies (Conv) and 90 robotic (Robot) surgeries. The patients in the Robot group received more neoadjuvant chemotherapy (*p* < 0.001) and radiotherapy (*p* < 0.001). More rectum surgeries were performed in the Robot group (*p* < 0.001) Table S3. Fewer patients had node invasion in the Robot group (*p* = 0.006). Surgery time was longer (*p* < 0.001), and more blood loss quantity was observed (*p* < 0.001) in the Robot group. The patients in the Robot group received more remifentanil (*p* < 0.001) and less local anesthetic infiltration (*p* = 0.03) during the intraoperative period. No difference between the groups was noticed in terms of complications and length of stay. The median 3-year OS was not significantly different between the Conv and Robot groups (543 days [288.5; 744.5] vs. 470.5 days [235.5; 897.5], *p* = 1). The Kaplan–Meier curves were not significantly different between the groups (log-rank test *p* = 0.5). The 3-year OS was 90.9% for the Conv group and 93.3% for the Robot group (*p* = 0.7). The median 3-year RFS was not significantly different between the Conv and Robot groups (375 days [266.5; 607] vs. 270 days [145; 457], *p* = 0.17). The Kaplan–Meier curves were not significantly different between the groups (log-rank test *p* = 0.2). The 3-year RFS was 81.8% for the Conv group vs. 75.6% for the Robot group (*p* = 0.31) Figure S3 and Table S4.

## 4. Discussion

This study investigated the oncological impact of surgical techniques and analgesic strategies on colorectal cancer outcomes through a propensity score-matched approach. Minimally invasive procedures, including laparoscopic and robotic surgeries, decreased the risk of blood loss and complications and shortened the length of stay. However, no surgical approach statistically improved 3-year OS and RFS after colorectal surgery despite the potentially relevant clinical impact (3-year OS 547 versus 318 days and 3-year RFS 435 versus 340.5 days).

Previously published studies aimed to compare the major and minor surgical approaches for colorectal cancer. The retrospective trial of Nazzal et al. concluded that laparoscopy had more short-term benefits compared to laparotomy procedures [12]. However, this study did not include a robotic surgery cohort, did not apply a propensity score matching strategy to avoid selection bias due to non-randomization, and did not evaluate long-term outcomes. Two retrospective studies and two meta-analyses also investigated the impact of laparoscopic and robotic surgeries after colorectal cancer and revealed opposite results. Robotic surgery could induce fewer complications and a shorter length of stay, particularly in older patients [30,31], and improve OS [31,32] compared to the laparoscopic approach. Conversely, the meta-analysis of Wang et al. found no differences between groups [33]. In the current study, the robotic approach did not decrease the rate of complications and length of stay and did not improve survival. The study was not designed to investigate robotic vs. laparoscopic surgery, and the number of included patients in both groups was limited. Robotic surgery was also responsible for longer surgery duration and more blood loss in our study. At the time of inclusion, robotic surgery had just been introduced at our center, and surgeons had not yet fully mastered the robotic technique, which requires specific training programs.

Our study found some predictor factors for OS and RFS. Age, ASA classification, pT3-T4, and adjuvant chemotherapy are expected to impact oncological outcomes, but our study revealed that postoperative pain, PCEA, and lidocaine infusion could also be predictor factors for OS and RFS. In line with this, preclinical research suggested that acute pain could favor recurrences through its immunosuppressive action on Natural Killer cells, a subgroup of lymphocytes able to recognize and kill tumor cells spontaneously [34,35,36]. Moreover, ample preclinical evidence has demonstrated that local anesthetics induce cytotoxic effects against cancer cells and may promote an antitumor immune response [13,14,15,16,17]. These aforementioned findings are supported by six retrospective clinical trials that observed better outcomes after the use of epidural in colorectal cancer surgery [18,19,20,21,22,23,24,25,26]. Two randomized controlled trials also demonstrated that epidural or local injection of lidocaine improves OS after colon or breast cancer resection [27,37]. The mechanisms by which local anesthetics improve survival are still under debate. Intravenous infusion of lidocaine ensures 100% bioavailability. Thus, intravenous lidocaine enables continuous perfusion of the tumor and can control circulating tumor cells during the intraoperative period. It is more difficult to believe in the direct cytotoxic effect of local anesthetics through neuraxial anesthesia. The epidural is very far from the tumor site, and plasma concentrations of local anesthetics after epidural administration are very low. However, the epidural perfectly controls the glucocorticoid immunosuppressive stress generated by the surgery and could support the immune system to control residual tumor cells [38]. Volatiles, intravenous hypnotics, and opioids could also impact oncological outcomes. However, only three patients received intravenous hypnotics in our study, and whether opioids and volatiles promote antitumor or protumor effects remains unclear [39,40,41,42,43,44,45]. Therefore, we did not select these agents for multivariate analyses.

According to the anesthetic protocol at our center, all the patients received remifentanil and morphine during the perioperative period. Opioids remain the most effective analgesic for surgical pain. However, some preclinical studies have hypothesized that opioids could be potential protumor agents [46,47]. The challenge is to determine the optimal dose of opioids to perfectly control immunosuppressive pain without inducing the proliferation of tumor cells. We, therefore, investigated whether high-dose opioids could negatively influence OS and RFS. As no threshold of opioids was determined in the literature, we defined “high dose” as the highest 3rd IQR of opioids between both groups (i.e., remifentanil > 2100 µg, intraoperative morphine > 10 mg, and total intra- and postoperative morphine > 145 mg OME). Of note, open surgery is a very painful approach. PCEA represents the optimal technique to control severe pain. Thus, more patients in the Laparo group received PCEA, which was started during the surgery and allowed up to five postoperative days (if necessary) according to the protocol of our center. In consequence, less remifentanil and morphine IV were required during and after the procedure in the Laparo group compared to the Mini group. The use of high doses of remifentanil and morphine could negatively impact the outcome of the Mini group, while this surgery is less painful. It appears that surgical approaches and anesthesia protocols might act as intertwined factors that could influence outcomes after cancer surgery.

Our research has some limitations. Our study is a single-center retrospective observational trial. Only a few patients were included in the study after matching. Only information on death and recurrence 3 years after surgery was available. Finally, the generalization of the current findings is limited due to the absence of other centers.

## 5. Conclusions

In conclusion, our study suggests that minimally invasive procedures, including laparoscopic and robotic approaches, are safe and have better benefits for colorectal tumor removal compared to open procedures in terms of complications, blood loss, and length of stay. Prospective multicenter randomized controlled trials are now expected to confirm these findings.

## Figures and Tables

**Table 1:** Baseline characteristics of cohorts before and after matching.

	Before Matching				After Matching			
	Laparo (74)	Mini (211)	SMD	*p*-Value	Laparo (68)	Mini (68)	SMD	*p*-Value
**Demographic data**								
Female, *n* (%)	26 (35.1)	103 (48.8)	**0.280**	**0.06**	24 (35.3)	23 (33.8)	0.031	1
Age (mean (SD))	64.20 (12.66)	62.69 (13.77)	0.114	0.39	66.5 [54.75; 70.25]	61.5 [53; 71.25]	0.173	0.32
Age > 70 years old, *n* (%)	20 (27)	59 (28)	0.021	1	18 (26.5)	18 (26.5)	<0.001	1
BMI, kg/m^2^ (mean (SD))	26.34 (5.07)	25.37 (4.32)	0.207	0.14	26.18 [23.41; 29.06]	25.74 [22.66; 27.69]	0.066	0.59
BMI > 25 kg/m^2^, *n* (%)	43 (58.1)	91 (43.1)	**0.303**	**<0.001**	38 (55.9)	38 (55.9)	<0.001	1
ASA 3-4, *n* (%)	14 (18.9)	26 (12.3)	0.182	0.23	13 (19.1)	10 (14.7)	0.118	0.65
Tobacco, *n* (%)	27 (36.5)	55 (26.1)	0.226	0.12	25 (36.8)	19 (27.9)	0.189	0.36
Alcohol, *n* (%)	12 (16.2)	20 (9.5)	0.202	0.17	9 (13.2)	8 (11.8)	0.044	1
Undernutrition, *n* (%)	20 (27)	41 (19.4)	0.181	0.23	19 (27.9)	18 (26.5)	0.033	1
**History**								
Stroke, *n* (%)	1 (1.4)	8 (3.8)	0.155	0.45	1 (1.5)	1 (1.5)	<0.001	1
Hypertension, *n* (%)	27 (36.5)	63 (29.9)	0.141	0.36	23 (33.8)	22 (32.4)	0.031	1
Diabetes, *n* (%)	12 (16.2)	26 (12.3)	0.112	0.52	11 (16.2)	16 (23.5)	0.185	0.39
COPD, *n* (%)	4 (5.4)	4 (1.9)	0.188	0.21	4 (5.9)	0 (0)	0.354	0.12
Cardiopathy, *n* (%)	3 (4.1)	9 (4.3)	0.011	1	3 (4.4)	4 (5.9)	0.067	1
Cardiac failure, *n* (%)	2 (2.7)	1 (0.5)	0.179	0.17	2 (2.9)	0 (0)	0.246	0.5
NYHA			0.198				0.289	
I, *n* (%)	54 (73)	149 (70.6)		0.81	48 (70.6)	44 (64.7)		0.58
II, *n* (%)	15 (20.3)	55 (26.1)		0.40	15 (22.1)	22 (32.4)		0.25
III, *n* (%)	5 (6.8)	7 (3.3)		0.35	5 (7.4)	2 (2.9)		0.44
Kidney insufficiency, *n* (%)	3 (4.1)	8 (3.8)	0.014	1	2 (2.9)	3 (4.4)	0.078	1
Family history of CRC, *n* (%)	8 (10.8)	30 (14.2)	0.103	0.59	7 (10.3)	9 (13.2)	0.091	0.79
**Medication**								
Statin, *n* (%)	8 (10.8)	40 (19)	0.230	0.15	8 (11.8)	15 (22.1)	0.277	0.17
Beta-blocker, *n* (%)	7 (9.5)	29 (13.7)	0.134	0.46	7 (10.3)	9 (13.2)	0.091	0.79
ACEi/ARB, *n* (%)	15 (20.3)	38 (18)	0.057	0.80	11 (16.2)	17 (25)	0.220	0.29
Aspirin, *n* (%)	8 (10.8)	25 (11.8)	0.033	0.98	8 (11.8)	8(11.8)	<0.001	1
Opiate analgesics, *n* (%)	2 (2.7)	10 (4.7)	0.108	0.74	1 (1.5)	3 (4.4)	0.175	0.62
**ERAS**, *n* (%)	2 (2.7)	4 (1.9)	0.054	0.65	2 (2.9)	1 (1.5)	0.100	1
**Neoadjuvant therapy**								
Chemotherapy, *n* (%)	33 (44.6)	53 (25.1)	**0.418**	**<0.003**	30 (44.1)	31 (45.6)	0.030	1
Radiotherapy, *n* (%)	28 (37.8)	56 (26.5)	0.244	0.09	26 (38.2)	31 (45.6)	0.149	0.49
**Biology**								
Creatinine, µmol/L (mean (SD))	73.36 (32.63)	71.03 (21.5)	0.085	0.57	70 [59; 79.25]	67 [59; 80.25]	0.010	0.94
Albumin, g/L (mean (SD))	34.34 (6.45)	33.08 (7.08)	0.186	0.16	34 [29; 40]	36.5 [29; 41]	0.022	0.99
Leukocytes, G/L (mean (SD))	8.29 (6.90)	7.72 (3.14)	0.106	0.49	6.8 [5.48; 9.18]	7.2 [5.8; 9.5]	0.119	0.64
Hb, g/dL (mean (SD))	12.46 (3.60)	12.45 (2.08)	0.003	0.98	12.10 [11; 13.53]	12.9 [11.3; 14]	0.049	0.13
CEA, mg/L (median [IQR])	5.6 [2; 79]	6.10 [2; 79.7]	0.218	0.50	6 [2; 79]	4.3 [1.5; 79]	0.061	0.46
**Tumor site**			0.254				0.490	
Left colon, *n* (%)	32 (43.2)	85 (40.3)		0.76	30 (44.1)	25 (36.8)		0.48
Right colon, *n* (%)	12 (16.2)	33 (15.6)		1	11 (16.2)	6 (8.8)		0.30
Transverse colon, *n* (%)	4 (5.4)	3 (1.4)		0.21	3 (4.4)	0 (0)		0.24
Rectum, *n* (%)	26 (35.1)	90 (42.7)		**<0.001**	24 (35.3)	37 (54.4)		0.04
**TNM**								
T3-T4 (vs. T1-T2), *n* (%)	33 (44.6)	63 (29.9)	**0.308**	**0.03**	30 (44.1)	29 (42.6)	0.030	1
N+ (vs. N0), *n* (%)	41 (55.4)	148 (70.1)	**0.308**	**0.006**	39 (57.4)	37 (54.4)	0.059	0.86

Bold values indicate statistically significant differences. Abbreviations: ACEi, angiotensin-converting enzyme inhibitors; ARB, angiotensin II receptor blockers; ASA, American Society of Anesthesiology scale; BMI, body mass index; CEA, carcinoembryonic antigen; COPD, chronic obstructive pulmonary disease; CRC, colorectal cancer; Hb, hemoglobin; ERAS, enhanced recovery after surgery; IQR, interquartile range; NYHA, the New York Heart Association; TNM, tumor node metastasis stage.

**Table 2:** Intraoperative and postoperative variables before and after matching.

	Before Matching				After Matching			
	Laparo (74)	Mini (211)	SMD	*p*-Value	Laparo (68)	Mini (68)	SMD	*p*-Value
**pTNM**								
pT3-T4 (vs. pT1-T2), *n* (%)	49 (66.2)	135 (64)	0.047	0.84	45 (66.2)	37 (54.4)	0.242	0.22
pN+ (vs. pN0), *n* (%)	19 (25.7)	72 (34.1)	0.185	0.23	17 (25)	23 (30.8)	0.195	0.35
**R-category**			0.273				0.354	
R0, *n* (%)	70 (94.6)	209 (99.1)		0.07	64 (94.1)	68 (100)		0.12
R1, *n* (%)	2 (2.7)	0 (0)		0.07	2 (2.9)	0 (0)		0.49
R2, *n* (%)	2 (2.7)	2 (0.9)		0.28	2 (2.9)	0 (0)		0.49
**Microscopic resection margin**			0.211				0.398	
0–1 mm, *n* (%)	3 (4.1)	3 (1.4)		0.18	3 (4.4)	0 (0)		0.24
1–2 mm, *n* (%)	2 (2.7)	2 (0.9)		0.28	2 (2.9)	0 (0)		0.49
>2 mm, *n* (%)	69 (93.2)	206 (97.6)		0.16	63 (92.6)	68 (100)		0.06
**Surgery**								
Duration, min (mean (SD))	268.95 (112.83)	261.79 (103.99)	0.066	0.86	260.5 [205.5; 326.5]	251 [179; 331]	0.110	0.47
Blood loss, ml (median [IQR])	60 [0; 300]	0 [0; 55]	0.504	**<0.001**	75 [0; 350]	0 [0; 85]	0.554	**<0.001**
Transfusion, *n* (%)	6 (8.1)	7 (3.3)	0.208	0.17	6 (8.8)	3 (4.4)	0.178	0.49
**Anesthesia**								
TIVA (vs. volatile), *n* (%)	2 (2.7)	1 (0.5)	0.179	0.17	2 (2.9)	1 (1.5)	0.100	1
Remifentanil, µg (median [IQR])	1047 [560.2; 1573]	1265 [914; 1854]	0.162	**<0.01**	1047 [627.8; 1589]	1424 [960; 2107]	0.239	**0.02**
Lidocaine infusion, *n* (%)	23 (31.1)	140 (66.4)	0.754	**<0.001**	21 (30.9)	40 (58.8)	0.585	**0.002**
Ketamine infusion, *n* (%)	65 (87.8)	182 (86.3)	0.047	0.89	61 (89.7)	58 (85.3)	0.134	0.6
PCEA, *n* (%)	43 (58.1)	17 (8.1)	1.256	**<0.001**	39 (57.4)	7 (10.3)	1.147	**<0.001**
TAP block, *n* (%)	5 (6.8)	3 (1.4)	0.272	**0.03**	5 (7.4)	2 (2.9)	0.201	0.44
Infiltration, *n* (%)	2 (2.7)	25 (11.8)	0.358	**0.02**	1 (1.5)	6 (8.8)	0.337	0.12
Acetaminophen, *n* (%)	71 (95.9)	210 (99.5)	0.242	0.06	65 (95.6)	67 (98.5)	0.175	0.62
Nefopam, *n* (%)	63 (85.1)	196 (92.9)	0.250	0.08	58 (85.3)	60 (88.2)	0.087	0.8
Tramadol, *n* (%)	49 (66.2)	122 (57.8)	0.174	0.26	45 (66.2)	41 (60.3)	0.122	0.59
Morphine IV, mg (median [IQR])	0 [0; 9.75]	8 [6; 10]	0.618	**<0.001**	0 [0; 10]	7.5 [5; 10]	0.502	**0.001**
**Postoperative period**								
Acetaminophen, *n* (%)	70 (94.6)	202 (95.7)	0.053	0.75	64 (94.1)	64 (94.1)	<0.001	1
Nefopam, *n* (%)	61 (82.4)	149 (70.6)	0.282	0.07	57 (83.8)	55 (80.9)	0.077	0.82
Tramadol, *n* (%)	49 (66.2)	121 (57.3)	0.183	0.23	46 (67.6)	38 (55.9)	0.244	0.22
NSAID, *n* (%)	11 (14.9)	26 (12.3)	0.074	0.72	11 (16.2)	10 (14.7)	0.041	1
Total morphine intra- and postoperative OME, mg (median [IQR])	45 [0; 135.68]	52.50 [27; 117.45]	0.001	**0.04**	49.05 [0; 144.07]	69.30 [24; 140.47]	0.051	0.13
VAS POD1 (median [IQR])	3 [1; 4]	3 [2; 5]	0.245	0.07	3 [1; 4]	3 [2; 5]	0.135	0.35
VAS POD2 (median [IQR])	2 [1; 4]	2 [1; 4]	0.046	0.76	2 [1; 4]	2.5 [1; 4]	0.014	0.97
Chemotherapy, *n* (%)	38 (51.4)	105 (49.8)	0.032	0.89	35 (51.5)	37 (54.4)	0.059	0.86
*Chemotherapy*			0.370				0.289	
folfiri, *n* (%)	1 (1.4)	0 (0)		0.26	1 (1.5)	0 (0)		1
folfirinox, *n* (%)	0 (0)	1 (0.5)		1	0 (0)	0 (0)		1
folfox, *n* (%)	25 (33.8)	54 (25.6)		0.23	23 (33.8)	24 (35.3)		1
LV5FU2, *n* (%)	1 (1.4)	0 (0)		0.58	1 (1.5)	0 (0)		1
xeloda, *n* (%)	6 (8.1)	23 (10.9)		0.65	5 (7.4)	5 (7.4)		1
xelox, *n* (%)	5 (6.8)	27 (12.8)		0.23	5 (7.4)	8 (11.8)		0.40
Time between surgery and chemotherapy, *d* (median [IQR])	39 [34; 57.50]	45 [39; 56]	0.207	0.41	39 [34.5; 56.75]	47 [40; 57]	0.148	0.42
Immunotherapy, *n* (%)	0 (0)	1 (0.5)	0.098	1	0 (0)	0 (0)	<0.001	1
CEA at 3 years, mg/L (median [IQR])	1.9 [0.72; 2.36]	1.8 [0.66; 2]	0.160	0.49	1.77 [0.56; 2.32]	1.8 [0.6; 2]	0.227	0.59
Length of stay, *d* (median [IQR])	12 [8.25; 19]	8 [5.5; 11]	0.567	**<0.001**	12 [8.75; 19]	8 [7; 12.25]	0.342	**<0.001**
**Complications**								
> or =1 complication at POD7, *n* (%)	23 (31.1)	34 (16.1)	0.358	**0.009**	22 (32.4)	16 (23.5)	0.198	0.34
Clavien-Dindo at POD7 major (3b-5), *n* (%)	6 (8.1)	9 (4.3)	0.160	0.33	5 (7.4)	3 (4.4)	0.125	0.72
> or =1 complication at 3 months, *n* (%)	23 (31.1)	15 (7.1)	0.640	**<0.001**	20 (29.4)	8 (11.8)	0.447	**0.02**
Clavien-Dindo at 3 months major (3b-5), *n* (%)	15 (20.3)	11 (5.2)	0.464	**<0.001**	12 (17.6)	7 (10.3)	0.213	0.32
Recurrence at 3 years, *n* (%)	15 (20.3)	44 (20.9)	0.014	1	13 (19.1)	14 (20.6)	0.037	0.83
Local recurrence, *n* (%)	3 (4.1)	6 (2.8)	0.066	0.70	3 (4.4)	1 (1.5)	0.175	0.62
Node recurrence, *n* (%)	0 (0)	3 (1.4)	0.170	0.57	0 (0)	0 (0)	<0.001	1
Metastases, *n* (%)	12 (16.2)	35 (16.6)	0.010	1	10 (14.7)	13 (19.1)	0.118	0.65
Time between surgery and recurrence, *d* (median [IQR])	350.5 [134.5; 614]	308.5 [201; 578.2]	0.166	0.80	435 [185.5; 709]	340.5 [225; 483.5]	0.308	0.77
Death at 3 years, *n* (%)	9 (12.2)	18 (8.5)	0.119	0.36	6 (8.8)	4 (5.9)	0.113	0.74
Time between surgery and death, *d* (median [IQR])	331 [109; 840]	572.5 [284.2; 880.2]	0.262	0.47	547 [164.5; 820.8]	318 [273; 460.2]	0.292	0.91

Bold values = statistically significant difference. Abbreviations: CEA, carcinoembryonic antigen; IQR, interquartile range; IV, intravenous; NSAID, non-steroidal anti-inflammatory drugs; OME, oral morphine equivalents; PCEA, patient-controlled epidural analgesia; POD, postoperative day; TAP block, transversus abdominis plane block; TNM, tumor node metastasis stage; TIVA, total intravenous anesthesia; VAS, visual analog scale.

**Table 3:** Univariate and multivariate Cox Hazard Model for overall survival.

	Univariate Analysis			Multivariate Analysis		
	HR	95% CI	*p*-Value	HR	95% CI	*p*-Value
***Demographic variables***						
Age	1.1	[1–1.2]	**<0.01**	1.08	[1.02–1.15]	**<0.01**
Female	0.81	[0.21–3.1]	0.76			
ASA > 3	3.5	[0.99–12]	**0.05**	1.86	[0.44–7.74]	0.40
BMI > 25 kg/m^2^	1.2	[0.33–4.2]	0.80			
***Tumor***						
pT3-T4 (vs. pT1-pT2)	0.97	[0.27–3.4]	0.96	1.52	[0.37–6.26]	0.56
pN+ (vs. pN0)	0.6	[0.13–2.8]	0.51			
R1-2 (vs. R0)	<0.001	[0-inf]	1			
***Surgery***						
Mini (vs Laparo)	0.66	[0.19–2.3]	0.52	0.43	[0.11–1.72]	0.23
Surgical duration > 6 h	2.9	[0.81–10]	0.1			
Blood loss > 350 mL	1.2	[0.26–5.8]	0.79			
Transfusion	1.5	[0.19–12]	0.70			
***Intraoperative analgesia***						
Remifentanil > 2100 µg	1.6	[0.41–6.2]	0.50			
Tramadol	1.4	[0.35–5.3]	0.65			
Morphine > 10 mg (IV)	0.86	[0.18–4]	0.84			
PCEA	0.48	[0.1–2.3]	0.36	0.12	[0.02–0.86]	**0.03**
Lidocaine infusion	0.81	[0.23–2.9]	0.75	0.34	[0.08–1.57]	0.17
***Postoperative analgesia***						
NSAID	1.4	[0.3–6.6]	0.66			
Tramadol	0.6	[0.17–2.1]	0.42			
Total morphine at POD7 > 145 mg (OME)	2	[0.57–7.2]	0.27			
VAS > 3 within 72 h postoperative	1.6	[0.42–6.3]	0.47			
***Adjuvant chemotherapy***	0.21	[0.046–1]	**0.05**	0.27	[0.05–1.53]	0.14

Bold values = statistically significant difference. Abbreviations: ASA, American Society of Anesthesiology scale; BMI, body mass index; CI, confidence interval; HR, hazard ratio; IV, intravenous; NSAID, non-steroidal anti-inflammatory drugs; OME, oral morphine equivalents; PCEA, patient-controlled epidural analgesia, POD, postoperative day; VAS, visual analog scale.

**Table 4:** Univariate and multivariate Cox Hazard Model for recurrence-free survival.

	Univariate Analysis			Multivariate Analysis		
	HR	95% CI	*p*-Value	HR	95% CI	*p*-Value
***Demographic variables***						
Age	1	[0.98–1]	0.37			
Female	1.3	[0.61–2.8]	0.48			
ASA > 3	1.2	[0.45–3.1]	0.72			
BMI > 25 kg/m^2^	1.4	[0.63–3]	0.41			
***Tumor***						
pT3-T4 (vs. pT1-pT2)	2.5	[1–6.3]	**0.045**	2.17	[0.79–5.96]	0.13
pN+ (vs. pN0)	1.8	[0.83–3.9]	0.14	1.38	[0.55–3.48]	0.50
R1-R2 (vs. R0)	<0.001	[0-inf]	1			
***Surgery***						
Mini (vs. Laparo)	1.1	[0.5–2.3]	0.87	0.81	[0.32–2.04]	0.65
Surgical duration > 6 h	0.73	[0.25–2.1]	0.56			
Blood loss >350 mL	1.8	[0.76–4.3]	0.18			
Transfusion	0.5	[0.07–3.7]	0.50			
***Intraoperative analgesia***						
Remifentanil > 2100 µg	1.1	[0.42–2.6]	0.92			
Tramadol	0.97	[0.44–2.1]	0.93			
Morphine > 10 mg (IV)	0.4	[0.12–1.3]	0.14			
PCEA	0.67	[0.28–1.6]	0.35	0.40	[0.13–1.28]	0.12
Lidocaine infusion	0.59	[0.26–1.3]	0.19	0.39	[0.16–0.97]	**0.04**
***Postoperative analgesia***						
NSAID	0.43	[0.1–1.8]	0.25			
Tramadol	1.4	[0.63–3.3]	0.38			
Total morphine at POD7 > 145 mg (OME)	1	[0.44–2.5]	0.93			
VAS > 3 within 72 h postoperative	2.7	[1.1–6.6]	**0.03**	2.29	[0.89–5.90]	0.08
***Adjuvant chemotherapy***	1.6	[0.72–3.4]	0.25	0.85	[0.33–2.14]	0.72

Bold values = statistically significant difference. Abbreviations: ASA, American Society of Anesthesiology scale; BMI, body mass index; HR, hazard ratio; IV, intravenous; NSAID, non-steroidal anti-inflammatory drugs; OME, oral morphine equivalents; PCEA, patient-controlled epidural analgesia, POD, postoperative day; VAS, visual analog scale.

## Data Availability

All analyzed data are included in the study and Supplemental Material. Further data are available upon request from the corresponding author upon reasonable request.

## Supplementary Materials

The following supporting information can be downloaded at https://www.mdpi.com/article/10.3390/medicina60081362/s1. Figure S1. Three-year OS and RFS between laparotomy and minimally invasive surgery (laparoscopic and robotic surgery) after colon cancer removal. Table S1. Univariate and multivariate Cox Hazard Model for overall survival after colon cancer surgery. Table S2. Univariate and multivariate Cox Hazard Model for recurrence-free survival after colon cancer surgery. Figure S2. Three-year OS and RFS between laparotomy and minimally invasive surgery (laparoscopic and robotic surgery) after rectum cancer removal. Figure S3. Three-year OS and RFS between conventional laparoscopy (Conv) and robotic surgery (Robot) for colorectal cancer removal. Table S3. Baseline characteristics between conventional laparoscopy (Conv) and robotic surgery (Robot). Table S4. Intraoperative and postoperative variables for conventional laparoscopy (Conv) and robotic surgery (Robot).

## References

[1] World Health Organization Colorectal Cancer. https://www.who.int/news-room/fact-sheets/detail/colorectal-cancer#:~:text=Colon%20cancer%20is%20the%20second,estimated%20to%20have%20occurred%20worldwide.

[2] Rentsch M., Schiergens T., Khandoga A., Werner J. (2016). Surgery for Colorectal Cancer—Trends, Developments, and Future Perspectives. Visc. Med..

[3] Finnerty C.C., Mabvuure N.T., Ali A., Kozar R.A., Herndon D.N. (2013). The surgically induced stress response. JPEN J. Parenter. Enter. Nutr..

[4] Yang H., Xia L., Chen J., Zhang S., Martin V., Li Q., Lin S., Chen J., Calmette J., Lu M. (2019). Stress-glucocorticoid-TSC22D3 axis compromises therapy-induced antitumor immunity. Nat. Med..

[5] Petrik J., Verbanac D., Fabijanec M., Hulina-Tomaskovic A., Ceri A., Somborac-Bacura A., Petlevski R., Grdic Rajkovic M., Rumora L., Kruslin B. (2022). Circulating Tumor Cells in Colorectal Cancer: Detection Systems and Clinical Utility. Int. J. Mol. Sci..

[6] Tsuchiya Y., Sawada S., Yoshioka I., Ohashi Y., Matsuo M., Harimaya Y., Tsukada K., Saiki I. (2003). Increased surgical stress promotes tumor metastasis. Surgery.

[7] Jacobs M., Verdeja J.C., Goldstein H.S. (1991). Minimally invasive colon resection (laparoscopic colectomy). Surg. Laparosc. Endosc..

[8] Schiedeck T.H., Schwandner O., Baca I., Baehrlehner E., Konradt J., Kockerling F., Kuthe A., Buerk C., Herold A., Bruch H.P. (2000). Laparoscopic surgery for the cure of colorectal cancer: Results of a German five-center study. Dis. Colon. Rectum.

[9] Hamabe A., Takemasa I., Kotake M., Nakano D., Hasegawa S., Shiomi A., Numata M., Sakamoto K., Kimura K., Hanai T. (2024). Feasibility of robotic-assisted surgery in advanced rectal cancer: A multicentre prospective phase II study (VITRUVIANO trial). BJS Open.

[10] Hahnloser D., Rrupa D., Grass F. (2023). Feasibility of on-demand robotics in colorectal surgery: First cases. Surg. Endosc..

[11] Matsuda T., Yamashita K., Hasegawa H., Oshikiri T., Hosono M., Higashino N., Yamamoto M., Matsuda Y., Kanaji S., Nakamura T. (2018). Recent updates in the surgical treatment of colorectal cancer. Ann. Gastroenterol. Surg..

[12] Nazzal K., Hasan L., Alqaseer A., Abdulla H.A., Majed A.S., Abushwemeh M.A., Salman E.S., Arafa M., Jawad A. (2024). Laparoscopic Versus Open Surgery for Colorectal Cancers: Clinical and Pathological Outcomes from a Single Institution in Bahrain. Gulf J. Oncol..

[13] Bezu L., Wu Chuang A., Sauvat A., Humeau J., Xie W., Cerrato G., Liu P., Zhao L., Zhang S., Le Naour J. (2022). Local anesthetics elicit immune-dependent anticancer effects. J. Immunother. Cancer.

[14] Yin D., Liu L., Shi Z., Zhang L., Yang Y. (2020). Ropivacaine Inhibits Cell Proliferation, Migration and Invasion, Whereas Induces Oxidative Stress and Cell Apoptosis by circSCAF11/miR-145-5p Axis in Glioma. Cancer Manag. Res..

[15] Chen J.L., Liu S.T., Huang S.M., Wu Z.F. (2022). Apoptosis, Proliferation, and Autophagy Are Involved in Local Anesthetic-Induced Cytotoxicity of Human Breast Cancer Cells. Int. J. Mol. Sci..

[16] Abdelaatti A., Buggy D.J., Wall T.P. (2024). Local anaesthetics and chemotherapeutic agents: A systematic review of preclinical evidence of interactions and cancer biology. BJA Open.

[17] Wu Chuang A., Kepp O., Kroemer G., Bezu L. (2021). Direct Cytotoxic and Indirect, Immune-Mediated Effects of Local Anesthetics Against Cancer. Front. Oncol..

[18] Vogelaar F.J., Abegg R., van der Linden J.C., Cornelisse H.G., van Dorsten F.R., Lemmens V.E., Bosscha K. (2015). Epidural analgesia associated with better survival in colon cancer. Int. J. Color. Dis..

[19] Holler J.P., Ahlbrandt J., Burkhardt E., Gruss M., Rohrig R., Knapheide J., Hecker A., Padberg W., Weigand M.A. (2013). Peridural analgesia may affect long-term survival in patients with colorectal cancer after surgery (PACO-RAS-Study): An analysis of a cancer registry. Ann. Surg..

[20] Cummings K.C., Xu F., Cummings L.C., Cooper G.S. (2012). A comparison of epidural analgesia and traditional pain management effects on survival and cancer recurrence after colectomy: A population-based study. Anesthesiology.

[21] Zimmitti G., Soliz J., Aloia T.A., Gottumukkala V., Cata J.P., Tzeng C.W., Vauthey J.N. (2016). Positive Impact of Epidural Analgesia on Oncologic Outcomes in Patients Undergoing Resection of Colorectal Liver Metastases. Ann. Surg. Oncol..

[22] Gottschalk A., Ford J.G., Regelin C.C., You J., Mascha E.J., Sessler D.I., Durieux M.E., Nemergut E.C. (2010). Association between epidural analgesia and cancer recurrence after colorectal cancer surgery. Anesthesiology.

[23] Gupta A., Bjornsson A., Fredriksson M., Hallbook O., Eintrei C. (2011). Reduction in mortality after epidural anaesthesia and analgesia in patients undergoing rectal but not colonic cancer surgery: A retrospective analysis of data from 655 patients in central Sweden. Br. J. Anaesth..

[24] Wu H.L., Tai Y.H., Mandell M.S., Tsou M.Y., Yang S.H., Chen T.H., Chang K.Y. (2020). Effect of epidural analgesia on cancer prognosis after colon cancer resection: A single-centre cohort study in Taiwan. BMJ Open.

[25] Wurster E.F., Pianka F., Warschkow R., Antony P., Brenner T., Weigand M.A., Schmied B.M., Buchler M.W., Tarantino I., Ulrich A. (2019). Peridural analgesia does not impact survival in patients after colon cancer resection: A retrospective propensity score-adjusted analysis. Int. J. Color. Dis..

[26] Day A., Smith R., Jourdan I., Fawcett W., Scott M., Rockall T. (2012). Retrospective analysis of the effect of postoperative analgesia on survival in patients after laparoscopic resection of colorectal cancer. Br. J. Anaesth..

[27] Christopherson R., James K.E., Tableman M., Marshall P., Johnson F.E. (2008). Long-term survival after colon cancer surgery: A variation associated with choice of anesthesia. Anesth. Analg..

[28] Cuschieri S. (2019). The STROBE guidelines. Saudi J. Anaesth..

[29] Clavien P.A., Barkun J., de Oliveira M.L., Vauthey J.N., Dindo D., Schulick R.D., de Santibanes E., Pekolj J., Slankamenac K., Bassi C. (2009). The Clavien-Dindo classification of surgical complications: Five-year experience. Ann. Surg..

[30] Ammirati C.A., Passera R., Beltrami E., Peluso C., Francis N., Arezzo A. (2024). Laparoscopic and robotic surgery for colorectal cancer in older patients: A systematic review and meta-analysis. Minim. Invasive Ther. Allied Technol..

[31] Duhoky R., Rutgers M.L.W., Burghgraef T.A., Stefan S., Masum S., Piozzi G.N., Sagias F., Khan J.S. (2024). Long-Term Outcomes of Robotic Versus Laparoscopic Total Mesorectal Excisions: A Propensity-Score Matched Cohort study of 5-year survival outcomes. Ann. Surg. Open.

[32] Watanabe T., Sasaki K., Nozawa H., Murono K., Emoto S., Matsuzaki H., Yokoyama Y., Abe S., Nagai Y., Shinagawa T. (2024). Robotic Versus Laparoscopic Abdominoperineal Resection for Locally Advanced Rectal Cancer Following Preoperative Chemoradiotherapy. Vivo.

[33] Wang X., Ma R., Hou T., Xu H., Zhang C., Ye C. (2024). Robotic versus laparoscopic surgery for colorectal cancer in older patients: A systematic review and meta-analysis. Minim. Invasive Ther. Allied Technol..

[34] Sacerdote P., Manfredi B., Bianchi M., Panerai A.E. (1994). Intermittent but not continuous inescapable footshock stress affects immune responses and immunocyte beta-endorphin concentrations in the rat. Brain Behav. Immun..

[35] Page G.G., Ben-Eliyahu S., Yirmiya R., Liebeskind J.C. (1993). Morphine attenuates surgery-induced enhancement of metastatic colonization in rats. Pain.

[36] Fujisawa T., Yamaguchi Y. (1997). Autologous tumor killing activity as a prognostic factor in primary resected nonsmall cell carcinoma of the lung. Cancer.

[37] Badwe R.A., Parmar V., Nair N., Joshi S., Hawaldar R., Pawar S., Kadayaprath G., Borthakur B.B., Rao Thammineedi S., Pandya S. (2023). Effect of Peritumoral Infiltration of Local Anesthetic Before Surgery on Survival in Early Breast Cancer. J. Clin. Oncol..

[38] Carli F., Webster J., Pearson M., Pearson J., Bartlett S., Bannister P., Halliday D. (1991). Protein metabolism after abdominal surgery: Effect of 24-h extradural block with local anaesthetic. Br. J. Anaesth..

[39] Jeon S., Kim H.K., Kwon J.Y., Baek S.H., Ri H.S., Choi H.J., Cho H.R., Lee Y.S., Kim J.Y., Kim J. (2020). Role of Sevoflurane on Natural Killer Group 2, Member D-Mediated Immune Response in Non-Small-Cell Lung Cancer: An In Vitro Study. Med. Sci. Monit..

[40] Tabnak P., Masrouri S., Geraylow K.R., Zarei M., Esmailpoor Z.H. (2021). Targeting miRNAs with anesthetics in cancer: Current understanding and future perspectives. Biomed. Pharmacother..

[41] Enlund M., Berglund A., Enlund A., Lundberg J., Warnberg F., Wang D.X., Ekman A., Ahlstrand R., Flisberg P., Hedlund L. (2023). Impact of general anaesthesia on breast cancer survival: A 5-year follow up of a pragmatic, randomised, controlled trial, the CAN-study, comparing propofol and sevoflurane. eClinicalMedicine.

[42] Levi L., Hikri E., Popovtzer A., Dayan A., Levi A., Bachar G., Mizrachi A., Shoffel-Havakuk H. (2023). Effect of Opioid Receptor Activation and Blockage on the Progression and Response to Treatment of Head and Neck Squamous Cell Carcinoma. J. Clin. Med..

[43] Kuramochi T., Sano M., Kajiwara I., Oshima Y., Itaya T., Kim J., Ichimaru Y., Kitajima O., Masamune A., Ijichi H. (2024). Effects of tramadol via a micro-opioid receptor on pancreatic ductal adenocarcinoma in vitro and in vivo. Reg. Anesth. Pain Med..

[44] Yuval J.B., Lee J., Wu F., Thompson H.M., Verheij F.S., Gupta H.V., Irie T., Scarpa J.R., McCormick P.J., Smith J.J. (2022). Intraoperative opioids are associated with decreased recurrence rates in colon adenocarcinoma: A retrospective observational cohort study. Br. J. Anaesth..

[45] Wu H.L., Tai Y.H., Chang K.Y., Lin S.P. (2021). Dose-dependent association between morphine requirement and mortality risk after resection for hepatocellular carcinoma. Eur. J. Anaesthesiol..

[46] Khabbazi S., Hassanshahi M., Hassanshahi A., Peymanfar Y., Su Y.W., Xian C.J. (2019). Opioids and matrix metalloproteinases: The influence of morphine on MMP-9 production and cancer progression. Naunyn Schmiedebergs Arch. Pharmacol..

[47] Liu Z., Cheng S., Fu G., Ji F., Wang C., Cao M. (2020). Postoperative administration of ketorolac averts morphine-induced angiogenesis and metastasis in triple-negative breast cancer. Life Sci..

