# Supplementary material for: Impact of Surgical and Anesthetic Procedures after Colorectal Cancer Surgery: A Propensity Score-Matched Cohort Study (The PROCOL Study)
Source: Medicina (Kaunas). 2024 Aug 21;60(8):1362. doi: 10.3390/medicina60081362 (PMC11356255; doi:10.3390/medicina60081362)
Supplement: Supplementary file 1 [file medicina-60-01362-s001.zip › medicina-3131411-supplementary.pdf]

A

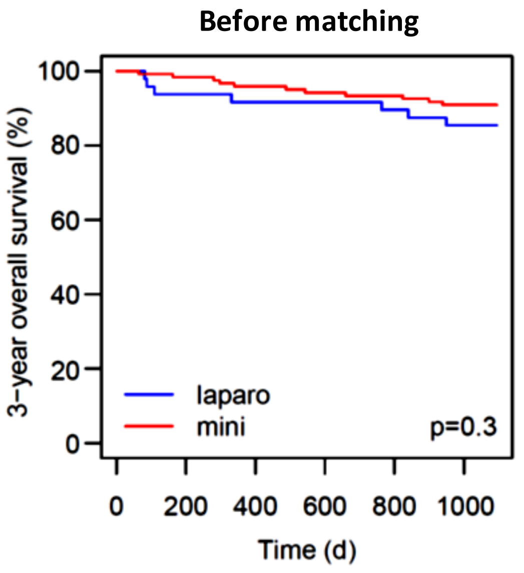

Time	0	200	400	600	800	1000	1095
Laparotomy	48	45	44	44	43	41	41
Minimally invasive surgery	121	119	116	114	113	110	110

B

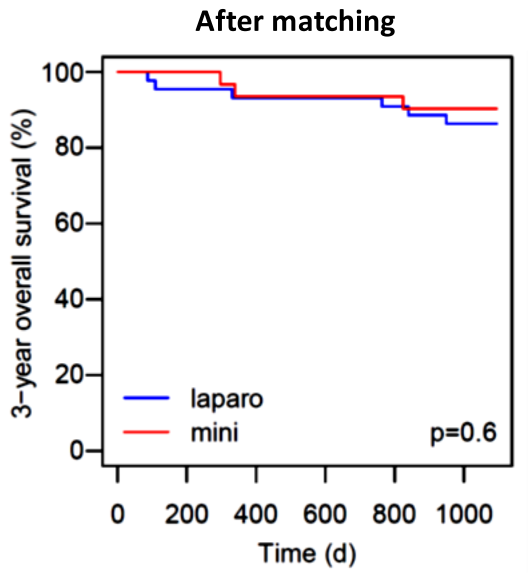

Time	0	200	400	600	800	1000	1095
Laparotomy	44	42	41	41	40	38	38
Minimally invasive surgery	31	31	29	29	29	28	28

C

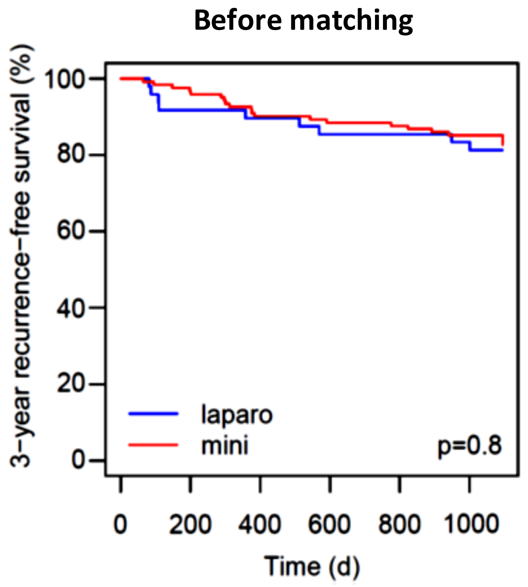

Time	0	200	400	600	800	1000	1095
Laparotomy	48	44	43	41	41	40	39
Minimally invasive surgery	121	117	109	107	106	103	100

D

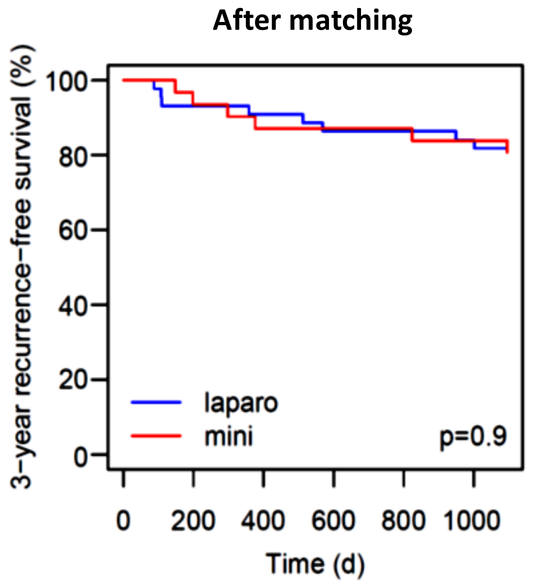

Time	0	200	400	600	800	1000	1095
Laparotomy	44	41	40	38	38	37	36
Minimally invasive surgery	31	29	27	27	27	26	25

Figure S1. Three-year OS and RFS between laparotomy and minimally invasive surgery (laparoscopic and robotic surgery) after colon cancer removal. A) Three-year OS before propensity score matching (log-rank test, *p*=0.3). **B)** Three-year OS after propensity score matching (log-rank test, *p*=0.6). **C)** Three-year RFS before propensity score matching (log-rank test, *p*=0.8). **D)** Three-year RFS after propensity score matching (log-rank test, *p*=0.9).

Table S1. Univariate and multivariate Cox Hazard Model for overall survival after colon cancer surgery

	**Univariate analysis**			**Multivariate analysis**		
	**Hazard Ratio**	**95% CI**	***p*-Value**	**Hazard Ratio**	**95% CI**	***p*-Value**
Demographic variables						
Age	1.1	[1-1.2]	**<0.01**	1.09	[1.03-1.17]	**<0.01**
Female	0.85	[0.21-3.4]	0.82			
ASA>3	2.3	[0.58-9.3]	0.23			
BMI>25 kg/m^2	1	[0.27-3.8]	0.99			
Tumor						
pT3-T4 (vs pT1-pT2)	0.8	[0.2-3.2]	0.75			
pN+ (vs pN0)	0.29	[0.04-2.3]	0.24			
R1-2 (vs R0)	<0.001	[0-inf]	1			
Surgery						
Mini vs laparo	0.7	[0.17-2.8]	0.61	0.35	[0.08-1.58]	0.17
Surgical duration>6h	3.7	[0.93-15]	0.06			
Blood loss>350mL	0.87	[0.11-7]	0.9			
Transfusion	1.4	[0.17-11]	0.78			
Intraoperative analgesia						
Remifentanil>2100µg	2.4	[0.51-12]	0.26			
Tramadol	1.1	[0.28-4.5]	0.88			
Morphine>10mg (IV)	1.1	[0.23-5.3]	0.91			
PCEA	0.42	[0.09-2]	0.27	0.14	[0.02-0.88]	**0.04**
Lidocaine infusion	0.92	[0.23-3.7]	0.9	0.64	[0.13-3.09]	0.58
Postoperative analgesia						
NSAID	1.3	[0.27-6.3]	0.74			
Tramadol	0.9	[0.24-3.4]	0.88			
Total morphine at POD7 >145mg (OME)	2.9	[0.77-11]	0.11			
VAS>3 within 72h postoperative	1.5	[0.37-5.9]	0.58			
Adjuvant chemotherapy	0.22	[0.05-1.1]	0.06	0.22	[0.04-1.12]	0.07

Bold values=statistically significant difference

Abbreviations: ASA, American society of anesthesiology scale; BMI, body mass index; CEA, carcinoembryonic antigen; CI, confidence interval; CRP, C-Reactive Protein; IV, intravenous; NSAID, non-steroidal anti-inflammatory drugs; OME, oral morphine equivalents; PCEA, patient-controlled epidural analgesia, POD, postoperative day; VAS, visual analog scale

Table S2. Univariate and multivariate Cox Hazard Model for recurrence-free survival after colon cancer surgery

	Univariate analysis			Multivariate analysis		
	**Hazard Ratio**	**95% CI**	***p*-Value**	**Hazard Ratio**	**95% CI**	***p*-Value**
Demographic variables						
Age	1	[0.99-1.1]	0.14			
Female	1.7	[0.61-4.9]	0.31			
ASA>3	1.3	[0.36-4.6]	0.71			
BMI>25 kg/m^2	2.1	[0.67-6.8]	0.2			
Tumor						
pT3-T4 (vs pT1-pT2)	2.6	[0.58-12]	0.21			
pN+ (vs pN0)	1.9	[0.64-5.4]	0.25			
R1-2 (vs R0)	<0.001	[0-inf]	1			
Surgery						
Mini vs laparo	1.1	[0.37-3]	0.92	0.57	[0.17-1.91]	0.36
Surgical duration>6h	2.1	[0.6-7.7]	0.24			
Blood loss>350mL	1.2	[0.27-5.4]	0.81			
Transfusion	0.82	[0.11-6.3]	0.85			
Intraoperative analgesia						
Remifentanil>2100µg	1.5	[0.33-6.6]	0.60			
Tramadol	0.72	[0.25-2.1]	0.55			
Morphine>10mg (IV)	0.62	[0.14-2.8]	0.54			
PCEA	0.38	[0.11-1.4]	0.14	0.21	[0.05-0.98]	**0.048**
Lidocaine infusion	0.74	[0.23-2.4]	0.62	0.37	[0.10-1.30]	0.12
Postoperative analgesia						
NSAID	0.32	[0.04-2.5]	0.28			
Tramadol	1.3	[0.44-3.9]	0.63			
Total morphine at POD7 >145mg (OME)	2	[0.68-6.1]	0.20			
VAS>3 within 72h postoperative	4.8	[1.1-21]	**0.04**	4.1	[0.88-19.11]	0.07
Adjuvant chemotherapy	1.1	[0.38-3.2]	0.86	0.83	[0.28-2.46]	0.74

Bold values=statistically significant difference

Abbreviations: ASA, American society of anesthesiology scale; BMI, body mass index; CEA, carcinoembryonic antigen; CI, confidence interval; CRP, C-Reactive Protein; IV, intravenous; NSAID, non-steroidal anti-inflammatory drugs; OME, oral morphine equivalents; PCEA, patient-controlled epidural analgesia, POD, postoperative day; VAS, visual analog scale

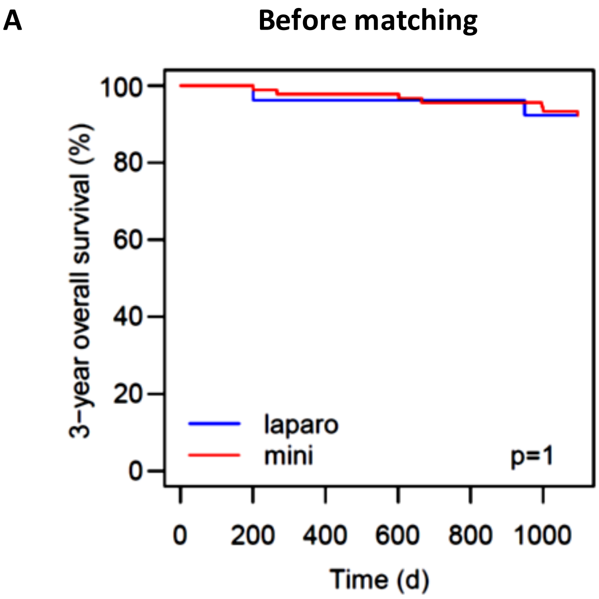

Time	0	200	400	600	800	1000	1095
Laparotomy	26	26	25	25	25	24	24
Minimally invasive surgery	90	90	88	88	86	84	83

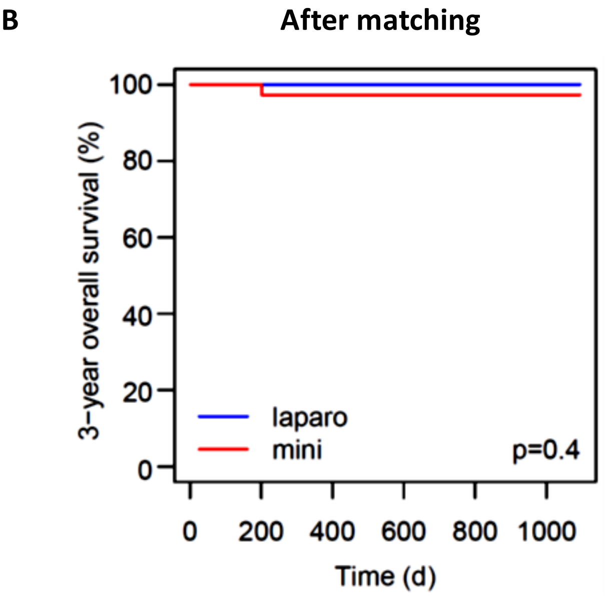

Time	0	200	400	600	800	1000	1095
Laparotomy	24	24	24	24	24	24	24
Minimally invasive surgery	37	36	36	36	36	36	36

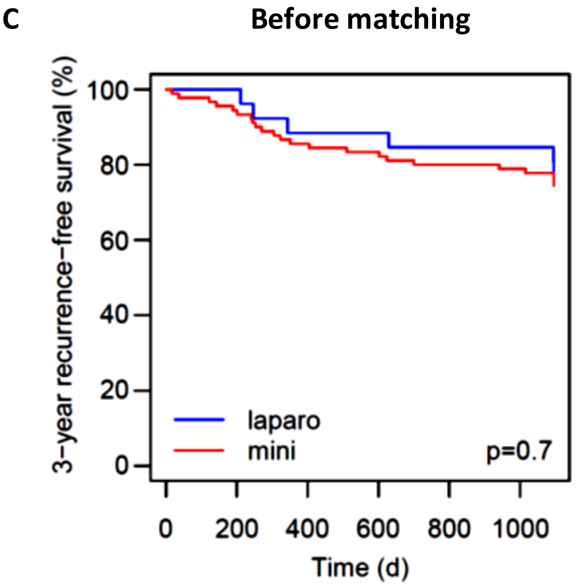

Time	0	200	400	600	800	1000	1095
Laparotomy	26	26	23	23	22	22	20
Minimally invasive surgery	90	85	77	75	72	71	67

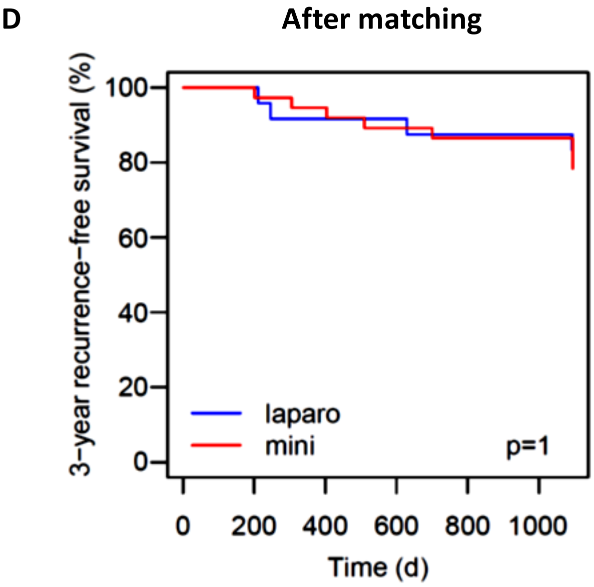

Time	0	200	400	600	800	1000	1095
Laparotomy	24	24	22	22	21	21	19
Minimally invasive surgery	37	37	35	33	32	32	29

Figure S2. Three-year OS and RFS between laparotomy and minimally invasive surgery (laparoscopic and robotic surgery) after rectum cancer removal. A) Three-year OS before propensity score matching (log-rank test, *p*=1). **B)** Three-year OS after propensity score matching (log-rank test, *p*=0.4). **C)** Three-year RFS before propensity score matching (log-rank test, *p*=0.7). **D)** Three-year RFS after propensity score matching (log-rank test, *p*=1).

A

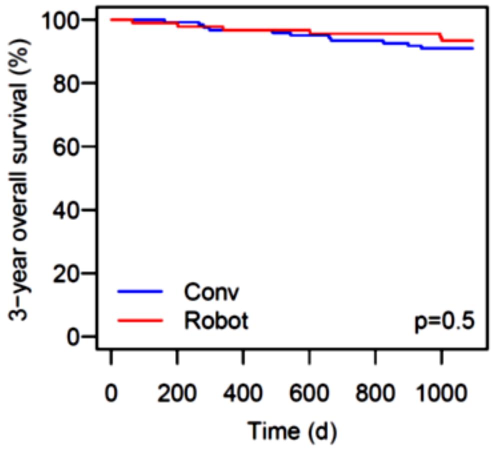

Time	0	200	400	600	800	1000	1095
Conv	121	120	117	115	113	110	110
Robot	90	89	87	87	86	85	84

B

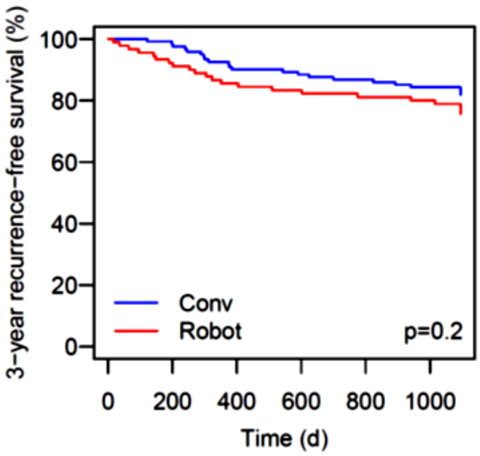

Time	0	200	400	600	800	1000	1095
Conv	121	119	109	107	105	102	99
Robot	90	83	77	75	73	72	68

Figure S3. Three-year OS and RFS between conventional laparoscopy (Conv) and robotic surgery (Robot) for colorectal cancer removal. A) Three-year OS (log-rank test, p=0.5). **B)** Three-year RFS (log-rank test, p=0.2).

Table S3. Baseline characteristics between conventional laparoscopy (Conv) and robotic surgery (Robot)

	**Conv. (121)**	**Robot. (90)**	***p*-Value**
**Demographic data**			
Female, *n* (%)	63 (52.1)	40 (44.4)	0.9
Age (mean (SD))	63.28 (13.91)	61.90 (13.61)	0.47
Age > 70 years old, *n* (%)	36 (29.8)	23 (25.6)	0.61
BMI kg/m² (mean (SD))	25.27 (4.30)	25.50 (4.37)	0.70
BMI>25kg/m², *n* (%)	55 (45.5)	37 (41.1)	0.62
ASA 3-4, *n* (%)	12 (9.9)	14 (15.6)	0.31
Tobacco, *n* (%)	26 (21.5)	29 (32.2)	0.11
Alcohol, *n* (%)	14 (11.6)	6 (6.7)	0.33
Undernutrition, *n* (%)	20 (16.5)	21 (23.3)	0.29
**History**			
Stroke, *n* (%)	4 (3.3)	4 (4.4)	0.73
Hypertension, *n* (%)	34 (28.1)	29 (32.2)	0.62
Diabetes, *n* (%)	10 (8.3)	16(17.8)	0.06
COPD, *n* (%)	3 (2.5)	1 (1.1)	0.64
Cardiopathy, *n* (%)	4 (3.3)	5 (5.6)	0.50
Cardiac failure, *n* (%)	1 (0.8)	0 (0)	1
NYHA			
I, *n* (%)	91(75.2)	58 (64.4)	0.12
II, *n* (%)	26(21.5)	29 (32.2)	0.22
III, *n* (%)	4 (3.3)	3 (3.3)	1
Kidney insufficiency, *n* (%)	2 (1.7)	6 (6.7)	0.08
Family history of CRC, *n* (%)	13 (10.7)	17 (18.9)	0.14
**Medication**			
Statins, *n* (%)	25 (20.7)	15 (16.7)	0.58
Beta-blocker, *n* (%)	17 (14)	12 (13.3)	1
ACEi/ARB, *n* (%)	20 (16.5)	18 (20)	0.64
Aspirin, *n* (%)	15 (12.4)	10 (11.1)	0.94
Opiate analgesics, *n* (%)	6 (5)	4 (4.4)	1
**ERAS**, *n* (%)	4 (3.3)	0 (0)	0.14
**Neoadjuvant therapy**			
Chemotherapy, *n* (%)	14 (11.6)	39 (43.3)	**<0.001**
Radiotherapy, *n* (%)	16 (13.2)	40 (44.4)	**<0.001**
**Biology**			
Creatinine, µmol (mean (SD))	69.63 (19.61)	72.91(23.80)	0.29
Albumin, g/L (mean (SD))	32.28 (6.99)	34.14 (7.10)	0.06
Leukocytes, G/L (median [IQR])	7 [5.9; 9.6]	6.85 [5.4; 9]	0.43
Hb, g/dL (mean (SD))	12.26 (2.28)	12.70 (1.77)	0.12
CEA, mg/L (median [IQR])	8.40 [2 ; 79.7]	6.10 [2 ; 79.7]	0.66
**Tumor site**			
Left colon, *n* (%)	60 (49.6)	25 (27.8)	**0.002**
Right colon, *n* (%)	29 (24)	4 (4.4)	**<0.001**
Transverse colon, *n* (%)	3 (2.5)	0 (0)	0.26
Rectum, *n* (%)	29 (24)	61 (67.8)	**<0.001**
**TNM**			
T3-T4 (vs T1-T2), *n* (%)	35 (28.9)	28 (31.1)	0.85
N+ (vs N0), *n* (%)	78 (64.5)	40 (44.4)	**0.006**

Bold values= statistically significant difference

Abbreviations: ACEi, angiotensin-converting enzyme inhibitors; ARB, angiotensin II receptor blockers; ASA, American society of anesthesiology scale; BMI, body mass index; CEA, carcinoembryonic antigen; COPD, chronic obstructive pulmonary disease; CRC, colorectal cancer; ERAS, enhanced recovery after surgery; IQR, interquartile range; Hb, hemoglobin; TNM, tumor node metastasis stage

Table S4. Intraoperative and postoperative variables for conventional laparoscopy (Conv) and robotic surgery (Robot)

	**Conv. (121)**	**Robot. (90)**	***p*-Value**
pT3-T4 (vs pT1-T2), *n* (%)	83 (68.6)	52 (57.8)	0.14
pN+ (vs N0), *n* (%)	15 (12.4)	5 (5.6)	0.15
**R-category**			
R0 (vs R1), *n* (%)	121 (100)	88 (97.8)	0.18
**Microscopic resection margin**			
>2 mm (vs <2 mm), *n* (%)	120 (99.2)	86 (95.6)	0.17
**Surgery**			
Surgical duration, min (mean (SD))	211.11 (68.36)	329.93 (105)	**<0.001**
Blood loss, mL (median [IQR])	0 [0 ; 0]	0 [0 ; 150]	**<0.001**
Transfusion, *n* (%)	6 (5)	1 (1.1)	
**Anesthesia**			
TIVA (vs volatiles), *n* (%)	1 (0.8)	0 (0)	1
Remifentanil, µg (mean (SD))	1202 (589.39)	1957.93 (1044.73)	**<0.001**
Lidocaine infusion, *n* (%)	74 (61.2)	66 (73.3)	0.09
Ketamine infusion, *n* (%)	101 (83.5)	81 (90)	0.25
PCEA, *n* (%)	7 (5.8)	10 (11.1)	0.25
TAP block, *n* (%)	3 (2.5)	0 (0)	0.26
Infiltration, *n* (%)	20 (16.5)	5 (5.6)	**0.03**
Acetaminophen, *n* (%)	120 (99.2)	90 (100)	1
Nefopam, *n* (%)	110 (90.9)	86 (95.6)	0.28
Tramadol, *n* (%)	71 (58.7)	51 (56.7)	0.31
Morphine, mg (mean (SD))	8.96 (4.96)	7.73 (4.51)	0.06
**Postoperative period**			
Acetaminophen, *n* (%)	115 (95)	87 (96.7)	0.74
Nefopam, *n* (%)	79 (65.3)	70 (77.8)	0.07
Tramadol, *n* (%)	73 (60.3)	48 (53.3)	0.38
NSAID, *n* (%)	16 (13.2)	10 (11.1)	0.80
Total morphine intra- and postoperative, mg OME (median [IQR])	48.30 [24 ; 111.30]	61.50 [30 ; 124.95]	0.24
VAS POD1 (median [IQR])	4 [2 ; 5]	3 [1.25 ; 5]	0.11
VAS POD2 (median [IQR])	3 [1 ; 4]	2 [1 ; 4]	0.66
Chemotherapy, *n* (%)	62 (51.2)	43 (47.8)	0.68
Chemotherapy			
folfiri, *n* (%)	0 (0)	0 (0)	1
folfirinox, *n* (%)	0 (0)	1 (1.1)	0.43
folfox, *n* (%)	34 (28.1)	20 (22.2)	0.42
LV5FU2, *n* (%)	0 (0)	0 (0)	1
xeloda, *n* (%)	11 (9.1)	12 (13.3)	0.45
xelox, *n* (%)	17 (14)	10 (11.1)	0.67
Time between surgery and chemotherapy, *d* (median [IQR])	45 [39.25 ; 53.5]	45 [36 ; 57]	0.83
Immunotherapy, *n* (%)	0 (0)	1 (1.1)	0.43
CEA at 3 years, mg/L (median [IQR])	1.8 [0.51 ; 2]	1.7 [1 ; 2]	0.88
Length of stay, *d* (mean (SD))	8.98 (8.57)	10.88 (7.05)	0.08

Table S4 (continued). Intraoperative and postoperative variables for conventional laparoscopy (Conv) and robotic surgery (Robot)

**Complications**			
> or = 1 complication at POD7, *n* (%)	19 (15.7)	15 (16.7)	1
Clavien-Dindo at POD7 major (3b-5), *n* (%)	3 (2.5)	6 (6.7)	0.17
> or = 1 complication at 3 months, *n* (%)	7 (5.8)	8 (8.9)	0.55
Clavien-Dindo at 3 months major (3b-5), *n* (%)	6 (5)	5 (5.6)	1
Recurrence at 3 years, *n* (%)	22 (18.2)	22 (24.4)	0.31
Local recurrence, *n* (%)	3 (2.5)	3 (3.3)	0.70
Node recurrence, *n* (%)	2 (1.7)	1 (1.1)	1
Metastases, *n* (%)	17 (14)	18 (20)	0.27
Time between surgery and recurrences, *d* (median [IQR])	375 [266.5 ; 607]	270 [145 ; 457]	0.17
Death at 3 years, *n* (%)	11 (9.1)	6 (6.7)	0.70
Time between surgery and death, *d* (median [IQR])	543 [288.5 ; 744.5]	470.5 [235.5 ; 897.5]	1

Bold values= statistically significant difference

Abbreviations: CEA, carcinoembryonic antigen; IQR, interquartile range; NSAID, non-steroidal anti-inflammatory drugs; OME, oral morphine equivalents; PCEA, patient-controlled epidural analgesia; POD, postoperative day; TAP block, transversus abdominis plane block; TIVA, total intravenous anesthesia; VAS, visual analog scale
